# Discrepancies of remote techno-tolerance due to COVID-19 pandemic within Arab middle-east countries

**DOI:** 10.1007/s43995-023-00026-0

**Published:** 2023-05-10

**Authors:** Muhannad A. Abu-Hashem, Adnan Gutub, Osama Salem, Mohd Khaled Shambour, Qusai Shambour, Mohammad Shehab, Ahmad Izzat, Mufda J. Alrawashdeh

**Affiliations:** 1 Department of Geomatics, Architecture and Planning Faculty, King Abdulaziz University, Jeddah, Saudi Arabia; 2 Computer Engineering Department, College of Computer and Information Systems, Umm Al-Qura University, Makkah, Saudi Arabia; 3 Educational Technology and E-Learning Department, College of Education, Umm Al-Qura University, Makkah, Saudi Arabia; 4 The Custodian of the Two Holy Mosques Institute for Hajj and Umrah Research, Umm Al-Qura University, Makkah, Saudi Arabia; 5 Software Engineering Department, Faculty of IT, Al-Ahliyya Amman University, Amman, Jordan; 6 Artificial Intelligence & Software Engineering Departments, Faculty of Information Technology, Aqaba University of Technology, Aqaba, Jordan; 7 Faculty of Engineering and Information Technology, Israa University, Gaza, Palestine; 8 Department of Mathematics, College of Sciences and Arts, Qassim University, Al-Ras, Kingdom of Saudi Arabia

**Keywords:** COVID-19, E-techno, Traditional and virtual classes, Students’ perceptions, ANOVA

## Abstract

The coronavirus disease (COVID-19) changed the world’s lifestyle switching many techno-services to be provided remotely instead of direct usual physical interactions between people. This study focused on university students’ perceptions of this virtual technology-engineering change as discrepancies to be analyzed. The research surveyed 777 different students from four Arab middle-east neighboring countries, with related demographics and specifications, expressing full remarkable experiences of e-technology in virtual as well as traditional performances helping reveal overall tolerance possibilities. The study adopted examination technology via ANOVA to test discrepancies within students' perceptions for overall multi-factors deep analysis classification. The exploration highlighted an interesting range of pros and cons remarks including feminism and economic effect as well as other cultural and engineering interaction issues, raising signals to essentially consider and mutually benefit from adequate future generation e-techno adaptation within the region.

## Introduction

COVID-19 pandemic changed the lifestyle of millions of people around the world including techno tolerance analysis [1]. Without warning, most worldwide universities have to engineer the e-techno utilization adaptation to virtual instead of traditional strategic philosophy [2]. Many universities have adopted blended technology and online engineering exams as an IT assessment tool in their e-techno attitude before the COVID-19 pandemic [3]. Such universities spent a lot of time and money on virtual techno infrastructure such as servers, e-learning, portal development, and engineeringly training lecturers and students on e-IT tools. These universities, mentioned above, do not face many obstacles and challenges in converting the entire professional life style to e-learning, because they are experienced in this techno-tolerance. In contrast, many other universities have adopted traditional techno tools such as whiteboards, markers, and LCD projectors. These universities faced many difficulties to convert to virtual platforms and several failed to do so. Furthermore, in some geographic regions of the world, many factors affect virtual performance, such as the availability of electrical power, the ability to access the Internet, and computer devices [4]. Accordingly, there are many challenges with using virtual classes during the COVID-19 pandemic, including lack of technical and communication equipment, limited teacher supervision of students [3], and insufficient basic knowledge of teachers and students about the use of ICTs [5]. Besides that, the culture of virtual learning is not applied as commonly as in-class teaching, i.e. in many countries around the world, which plays a major factor in the success or failure of this kind of learning [4].

**Fig. 1:**
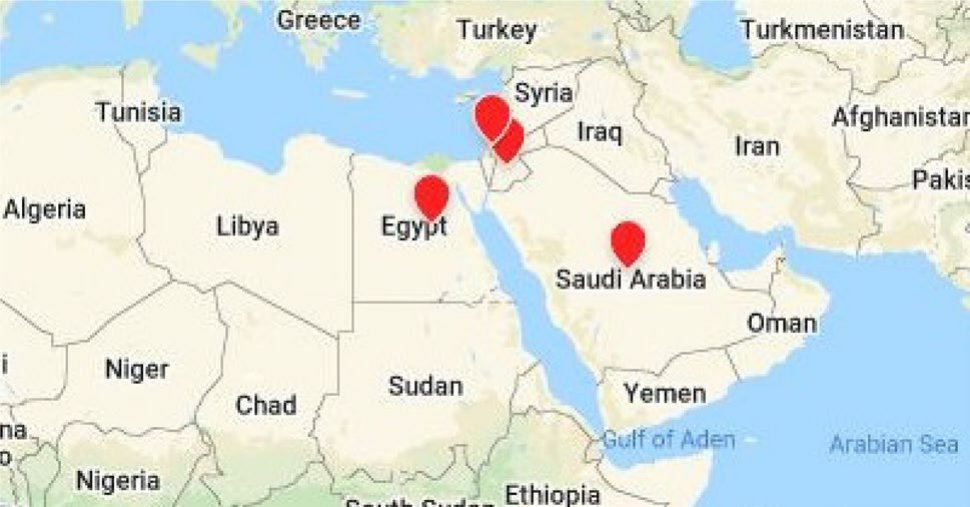
The countries of the Arab Middle East that were focused in this study

To gain some insight into the effectiveness of e-learning performance, we look at the results of a statistical survey gathered from 777 students from different countries, the majority (97.3%) belonging to Middle Eastern countries; Palestine, Jordan, Saudi Arabia, and Egypt, as the map shown in Fig. 1. The survey is obtained from various majors, including medical sciences/health, computer science and information technology, applied sciences, and Islamic sciences. The candidates’ degrees are undergraduate, diploma, master, and PhD. Moreover, the survey considers different devices that students use to keep up with virtual e-platforms such as mobile phones, laptops, and desktop computers similar to social media studies focus on Twitter [6]. The participation rate of males and females reached 46.5% and 53.5, respectively, different in philosophy than increasing participants’ study of Al-Shaarani [7].

Also, the results indicated that students in the fourth and fifth academic years face more problems in addressing the questions and gaining the required knowledge than other students during virtual sessions compared to traditional learning in the classroom. In fact, COVID-19 pandemic sternness driven remote e-learning suggesting this exploration as to aim spotting the students’ sensitivities regarding technology, i.e. as an approach for carrying the learning process during this pandemic worldwide problem as well as possibilities creating a new way of education norm. This investigation surveys of many students from dissimilar several countries concentrating on four Arab middle-east neighboring countries have been providing full attention statistical research recording numerous variations, though all are commonly culture related in demographics and specifications. The work expressed ANOVA noteworthy understandings of adopting e-technology in virtual and traditional concerts hoping to be serving the planning of education and its general tolerance future potentials. This work can further be assisting teaching management to focus on learning different options as well as looking for new methods and techniques to develop the progressions of practical schooling tuned for any future need.

The rest of the paper is structured as follows. Section 2 presents some of the related works covering background of the research. The material and study method are presented in Sect. 3. Section 4 provides results and discussion including sample data collection analysis and discussion. Finally, Sect. 5 concludes this paper by mentioning future research directions in this related field.

## Related works

Borstorff and Lowe [8] highlighted the importance of using distance learning technologies because of the availability of many instructional technology methods. Therefore, the researchers have become interested in reaching users’ satisfaction by enhancing and simplifying these methods. The authors took a sample consisting of 113 students from a Business College. The results showed that 88% of students were already using E-learning courses and 70% of students recommended using E-learning courses in the long term to their colleagues.

Relatively, Benigno [9] evaluation of online courses was the main contribution of his work. He focused on finding a mechanism to calculate the quantitative/qualitative assessment for participant performance and the learning process. Thus, a new approach has been introduced for small and medium e-techno institutions, which aims to evaluate the performance of online courses, and trained teachers and employees, as can further be linked to mobile computing survey analysis presented in Ref. [10].

Jayasinghe et al. [11] and Donovan et al. [12], evaluate the students’ performance by comparing online courses and traditional courses. For instance, Jayasinghe et al. [11] focused on studying and evaluating the students’ behavior by giving feedback on the provided paper exams for the students. It’s worth mentioning that there is no monitoring to evaluate the students’ performance by online techno mechanisms [13]. Therefore, the researchers have developed new methods for measuring the emotional level and behavior of the students using online techno performance systems. While Donovan et al. [12] conducted a study on a sample of 519 students who had a classroom course and an online course (same contents in both courses). The teachers evaluated the students’ performance and analyzed the answers in both courses. The results showed no significant changes in numerical ranks in the courses’ assessment forms.

COVID-19 pandemic motivated distance learning because of the severity of the virus spreading. In the medical field, distance learning becomes one of the main methods to teach residents and fellows [14]. Also, Abbasi et al. [15] conducted a current study for 377 students in the College of Medicine and Dentistry. The study aimed to observe students’ perceptions regarding e-learning as an approach for carrying the knowledge process during the pandemic of COVID-19. Unfortunately, the findings of the questionnaire illustrated that 77% of students didn’t prefer e-learning. Therefore, the administration should be looking for new methods and techniques to enhance the processes of distance learning, which motivated our research intention too.

Likewise, Aristovnik [16] conducted a large-scale study of more than 30 thousand students selected from 62 states to measure the effect of the COVID-19 pandemic. Although, there are not enough computer skills as well as anxiety and frustration regarding the epidemic and the restrictions imposed. However, most of the students' perspective was positive regarding the support provided by teachers and the methods that have been followed.

Al-Taweel [17] highlighted the academic perspective during the COVID-19 pandemic in multidisciplinary areas such as business administration, engineering, architecture, and design. The study recommends the following: the COVID-19 pandemic is not limited to the medical fields; all disciplines should work together to fight and stop the spread of this pandemic, and all universities should provide the needs for distance learning and look for alternative techniques to be ready for any future biological, economic, or natural crises.

Bui [18] prepared a dataset on the behavioral intent of female students regarding the use of videoconferencing tools in Vietnam during the COVID-19 pandemic. The dataset consists of 254 records using a 21-item survey that covers students’ perceptions of four topics, the COVID-19 context, computer playfulness, computer effectiveness, and behavioral intent to use video conferencing tools.

In another study, Khalil [19] explored undergraduate medical students’ perceptions regarding the effectiveness of virtual classes at Qassim university in Saudi Arabia. 60 students were engaged in eight focus group discussions. The analyzed interviews showed participants’ agreement that the virtual classes saved time and that their performance improved. Also, the results revealed that most pre-clinical students prefer virtual classes for the upcoming semesters. More recent research on students’ perceptions during the COVID-19 pandemic could be found in Ana [20] and Machado [21]. These studies triggered the need and showed some path to compare between related countries, Arab middle east neighbors in our case, in terms of accepting the technology as culture as well as accommodating its needs as available possibilities for proper e-education.

## Significance of study

The pandemic of COVID-19 has had a severe and wide-ranging impact on various aspects of everyday life [22–27]. Technology -at all levels- is one of the most severely impacted by the effects of COVID-19. During the pandemic, providers, educators, and students all suffer. They must figure out how to adjust to the sudden shift to virtual learning during a pandemic. However, there are numerous restrictions and practical issues regarding technological availability. Furthermore, students confront major learning challenges as a result of the loss of face-to-face learning, information exchange, and other factors. This study helps to highlight the student perceptions of the sudden shift to virtual learning during COVID-19 on the student adapting. Eight factors were chosen to represent a portion of students' virtual learning challenges, and these elements are discussed in the data section. On the other hand, this study focuses on developed countries, which suffer from many techno-tolerance challenges, which are mirrored in their students. Furthermore, This study investigates the factors that aid in determining the most critical factors that affected and confronted students during the COVID-19 pandemic. This will assist both the techno institutions and the students in dealing with the quick transition from traditional learning to virtual learning. Understanding the perspectives of students can aid in identifying the major challenges of shifting to virtual teaching at the undergraduate level. Once the concerns of students have been addressed, teachers and academic institutions can adapt their teaching methods and make decisions about the use of online technologies for virtual teaching purposes. Decision-makers can use the findings of this study’s analyses and recommendations to improve and develop the quality of techno services. This knowledge can make the transition to online teaching a lot easier.

## The materials and methods

**Fig. 2:**
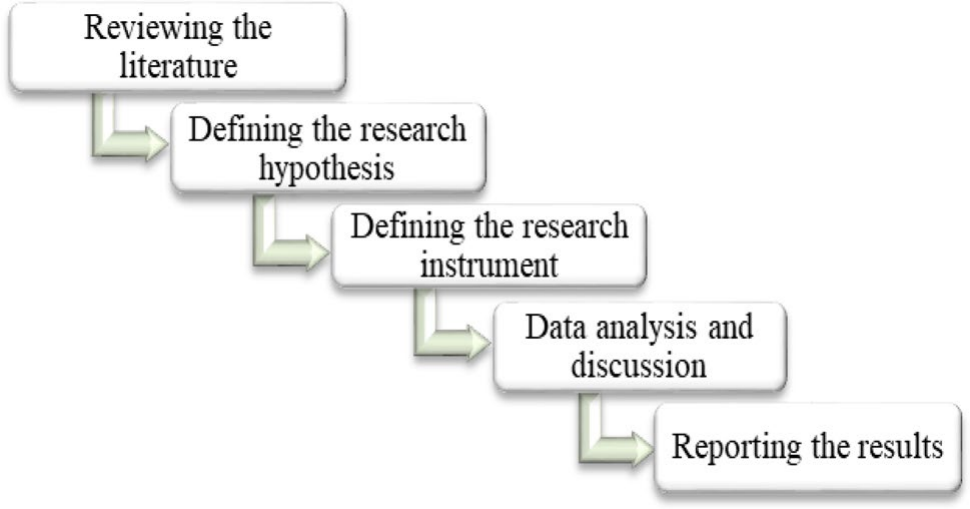
Research methodology approach

Figure 2 illustrates the methodology of the research, which includes a review of the field’s literature, the definition of the research hypothesis, the selection of an appropriate assessment tool for the ongoing research, statistical data analysis and discussion, and finally the presentation of the findings and results [22].

- Reviewing the Literature

Reviewing the literature is the first step in conducting any research. In this study, previous studies were discussed in the previous section which is directed to the students’ learning paradigms using virtual and traditional classes.

- Defining the research hypothesis

The hypothesis was defined as follows: Students’ perceptions of educational effectiveness between virtual and traditional teaching approaches cannot be restricted by traditional eight key factors, including gender, major, age, country, academic year level, academic degree, types of devices used, and whether, i.e. following old department/college offering virtual courses, prior to the outbreak of COVID-19.

- Defining the research instrument

To extract the students’ perceptions regarding the effectiveness of virtual learning over the traditional learning paradigms, a revised questionnaire was adopted from Borstorff and Lowe [8]. The questionnaire measures the student perceptions toward e-learning in the college environment consisting ten questions classified into five-dimension scales, including the interaction between students, classmates, and their instructors; communication problems with classmates and the instructor; the difficulty of course material and expectations of learning; and future courses in distance learning. The questionnaire was developed using the Google Form application and sent via social media to participants.

Table 1 presents the dimension scales and their corresponding questions adopted from [8]. The respondents are requested to express their perspectives on all questions using a five-point Likert-type scale, with “1” denoting strong disagreement and “5” denoting strong agreement.

- Data analysis and discussion

**Table 1:** Students’ perceptions questionnaire Borstorff and Lowe [8]

Dimension Scales	Variables	
Scale 1. Interaction between students, classmates and their instructors	1	It is critical to communicate with my lecturer
	2	Interacting with my peers in distance e-techno is really important to me
Scale 2. Communication problems with classmates and the instructor	3	I had no trouble interacting with my instructor when I was taking a distance e-techno course and had questions or concerns
	4	Throughout the semester, I had no trouble interacting with my classmates
Scale 3. Difficulty of course material and expectations of learning	5	I believe I learn just as much in a distance learning setting as I would in a classroom setting physically with an instructor
	6	Projects/assignments that require me to learn new things are my favorites
	7	In a remote e-techno process, I feel just as challenged as I would in a typical classroom
Scale 4. Future courses in distance learning	8	If asked, I would advise my classmates to take remote e-techno courses
	9	If given the chance, I would take another distance e-techno course
Scale 5. clarity of instructions in a distance learning environment	10	The directions are quite clear with the remote learning system

Statistical methods were applied to the collected data from students representing 777 responses to generate the study test outcomes. The descriptive features of the collected data were extracted based on the inference basis of theoretical distributions which examine the stated hypothesis and explore the students’ perceptions regarding the learning and teaching process using traditional and virtual classes (VC) paradigms. To do so, the statistical tool for analysis of variance (ANOVA) is used to examine the research hypotheses and observe the significant differences between students' perceptions regarding the effectiveness of virtual teaching and the eight factors mentioned earlier.

- Reporting the results

In this step, the analyzed data and results are deeply discussed and reported in the research with the hope of providing valuable points for researchers in various fields and interests in their future studies.

## Results discussion

### Collecting sample data

The collected data from the designed questionnaire was 777 records, during the period from 13-Jun-20 to 20-Sep-20, filled by students from different demographics and specifications that enrich the current study with different expectations and perceptions. All students had experience with traditional and virtual teaching systems.

This study defined eight factors: gender, major specification, age, country of teaching, covered e-learning courses before the COVID-19 pandemic, academic year, candidate degree, and the device used in virtual classes. The different aspects of these factors reveal the actual perceptions of students regarding the traditional and virtual classes during the pandemic of COVID-19.

### Data analysis and discussion

The students’ evaluations of virtual classes’ effectiveness compared to traditional classes were assessed using ANOVA test. Table 2 provides the overall statistical outcomes for students’ perceptions of the usefulness of virtual classes versus traditional in-class teaching based on the dimension scales.

**Table 2:** Statistical outcomes for Five-Likert Scale questions’ on students’ perceptions of virtual classes

Dimension Scales	Mean	Std.	Descriptor
D1: Interaction between students, classmates, and their instructors	3.90	0.88	Closer to agree than undecided
D2: Communication problems with classmates and the instructor	2.72	1.13	Closer to undecided than disagree
D3: Difficulty of course techno material and expectations of learning	3.00	0.85	No-preferences
D4: Future courses in distance learning	3.07	1.16	No-preferences
D5: Clarity of instructions in a distance learning environment	3.00	1.21	No-preferences

As shown in Table 2, based on the mean values of students’ responses on the five scales of the study, we find out that three scales out of five are no-preferences on virtual classes’ effectiveness compared to traditional classes (Difficulty of course material and expectations of learning scale, Future courses in distance learning scale, and clearness of guidelines in a distance learning environment scale). Meanwhile, the mean value of students’ responses on the interaction between students, classmates, and their instructor’s scale are closer to agree than undecided on distance e-techno effectiveness compared to traditional in-class performance. Finally, the mean value of students’ responses on communication problems with classmates and the instructor is closer to no-preferences than disagreeing on virtual e-techno efficacy compared to traditional in-class adaptation.

Furthermore, the mean value of students’ responses on the interaction between students, classmates, and their instructor’s scale highlight the importance of interactions during the virtual sessions in virtual courses between students and instructors as well as the interactions among students. Moreover, the mean value of students’ responses on communication problems with classmates and the instructor scale indicates the limitations of current technology to overcome the physical distances and matches the level of traditional in-class learning communication. In addition, the mean value of students’ responses on the difficulty of course material and expectations of learning scale shows and highlights the need to improve the virtual learning process to match the traditional in-class learning. Finally, the mean values of future courses in the distance learning scale and clarity of instructions in a virtual learning environment scale highlight the need to increase students’ awareness of virtual e-techno’s advantages and potential.

It is to be mentioned that this no-preferences descriptor (Table 2) is relating to the mean and standard deviation values of overall remarks looked at all together. This does not indicate any problem or disagreement within the data collection nor survey answering, however, it gives realistic uncertainty impression from the four question below. To be specific, D2 is about communication, which gives variety of implication based on the internet services, as mostly dependent on many direct and indirect country and governmental factors not relating to COVID pandemic, making our study applicable notes as no-preferences. Similarly, question D3 is about techno material which did not change much due to COVID. Likewise, D4 and D5 are respectively about future learning and clarity of instructions that are both showing neutral preference among the total general participants making it also undecided. These no-preferences observations guided the research to the necessity of covering other statistical questions and key factors measures details in order to present fruitful recommendations as disc discussed scattered throughout the paper.

Table 3 shows the analysis results of the gender-based ANOVA test’s outcomes, the P-values exceed 0.05 on three scales out of five; the difficulty of course material and expectations of learning scale, future courses in virtual learning scale, and clarity of instructions in a virtual learning environment scale. It shows no significant differences on these scales based on gender which supports the study hypothesis for these three scales. Furthermore, all the results are within the same category as the general statistical results shown in Table 2 (except female students’ mean value which is closer to strongly agree than agree on the interaction between students, classmates, and their instructor’s scale and female students’ mean value which is closer to undecided than disagreeing on clarity of instructions in a virtual learning environment scale).

**Table 3:** Gender-based ANOVA test’s outcomes

Dimension Scales	Gender	N	Mean	Std.	F-value	P-value
D1	Male	361	3.73	0.92	27.29	2.2E-07
	Female	416	4.05	0.81		
D2	Male	361	2.89	1.15	16.11	6.5E-05
	Female	416	2.57	1.11		
D3	Male	361	3.00	0.90	0.05	0.83
	Female	416	2.99	0.82		
D4	Male	361	3.11	1.22	0.86	0.36
	Female	416	3.04	1.11		
D5	Male	361	3.07	1.25	1.89	0.17
	Female	416	2.95	1.17		

Furthermore, significant differences in students’ perceptions of gender are detected in the Interaction between students, classmates, and their instructor’s scale [P = 2.25E-07] and the Communication problems with classmates and the instructor scale [P = 6.50E-05], as listed in Table 4. The mean value of females is closer to agree than strongly agree on virtual teaching effectiveness indicating that interactions between students and instructors and among students are at a high level of importance for students, especially females.

**Table 4:** Results of the ANOVA test based on major

Dimension Scales	Major	N	Mean	Std.	F-value	P-value
D1	Medical Sciences/Health	430	3.86	0.90	2.75	0.02
	Engineering Sciences	35	3.84	0.91		
	Computer Science and Information Technology	148	3.84	0.80		
	Applied Sciences	4	3.25	0.87		
	Islamic Sciences	31	3.94	1.15		
	Others	129	4.14	0.77		
D2	Medical Sciences/Health	430	2.64	1.16	2.83	0.02
	Engineering Sciences	35	3.19	1.14		
	Computer Science and Information Technology	148	2.68	1.09		
	Applied Sciences	4	3.50	0.71		
	Islamic Sciences	31	2.68	1.11		
	Others	129	2.91	1.08		
D3	Medical Sciences/Health	430	2.93	0.85	4.71	3.0E-04
	Engineering Sciences	35	3.12	0.74		
	Computer Science and Information Technology	148	2.89	0.83		
	Applied Sciences	4	4.17	0.58		
	Islamic Sciences	31	3.13	1.03		
	Others	129	3.22	0.85		
D4	Medical Sciences/Health	430	3.00	1.15	2.38	0.04
	Engineering Sciences	35	3.20	1.19		
	Computer Science and Information Technology	148	2.97	1.20		
	Applied Sciences	4	3.50	1.23		
	Islamic Sciences	31	3.24	1.26		
	Others	129	3.35	1.12		
D5	Medical Sciences/Health	430	2.85	1.20	5.59	4.6E-05
	Engineering Sciences	35	3.29	1.20		
	Computer Science and Information Technology	148	2.95	1.20		
	Applied Sciences	4	2.75	0.96		
	Islamic Sciences	31	3.35	1.25		
	Others	129	3.42	1.12		

Therefore, it is recommended (Table 4) to offer multiple options and tools that support and simplify the interaction process. Also, it is noticed that the mean value of females on clarity of instructions in a virtual learning environment scale is closer to no-preferences than disagree indicating that female students face more problems with addressing questions and interacting with instructors, as compared with male students during virtual sessions.

As shown in Table 5, on all scales, the P-value is less than 0.05, showing significant variations within each scale based on the major for students’ perceptions which contradicts the study hypothesis. Furthermore, the mean value results can be considered within the same category as the general statistical results given by Table 2 where all majors are closer to agree (except applied sciences students, who are closer to undecided) on the interaction between students, classmates, and their instructor’s scale. Also, all majors are closer to no-preferences (except applied sciences students, who are closer to agreeing) on communication problems with classmates and the instructor. Furthermore, all majors are closer to no-preferences on the clarity of instructions in a virtual learning environment.

**Table 5:** The outcomes of the ANOVA test based on age

Dimension Scales	Age	N	Mean	Std.	F-value	P-value
D1	Less than 21	367	3.94	0.90	1.57	0.18
	21–24	368	3.84	0.87		
	25–28	31	4.03	0.72		
	29–32	3	4.17	1.04		
	More 32	8	4.44	0.62		
D2	Less than 21	367	2.91	1.16	8.45	1.0E-06
	21–24	368	2.51	1.07		
	25–28	31	2.69	1.22		
	29–32	3	3.83	0.29		
	More 32	8	3.75	0.85		
D3	Less than 21	367	3.00	0.84	3.11	0.01
	21–24	368	2.96	0.85		
	25–28	31	3.00	1.02		
	29–32	3	3.89	0.51		
	More 32	8	3.87	0.84		
D4	Less than 21	367	3.13	1.14	4.84	7.3E-04
	21–24	368	2.96	1.16		
	25–28	31	3.31	1.26		
	29–32	3	4.50	0.50		
	More 32	8	4.31	0.84		
D5	Less than 21	367	3.16	1.19	7.76	4.0E-06
	21–24	368	2.80	1.17		
	25–28	31	3.13	1.36		
	29–32	3	4.67	0.58		
	More 32	8	4.13	1.13		

A significant difference is detected in students’ perceptions within their major on the interaction between students, classmates, and their instructor’s scale [P < 0.02] indicating that applied sciences students are less concerned with the importance of interactions in virtual classes as compared to the opinion of students in different majors. Furthermore, a significant difference is detected in students’ perceptions within the major on communication problems with classmates and the instructor scale [P < 0.02] indicating that applied sciences students face fewer problems related to communication during the virtual sessions as compared to the students in different majors. Moreover, a significant distinction is discovered in students’ perceptions within major on the difficulty of course material and expectations of learning scale [P = 3.05E-04] indicating that applied sciences students experience fewer problems related to learning and absorbing knowledge during the virtual sessions as compared to the students in different majors. In addition, a significant distinction is discovered in students’ perceptions within major on the future courses in virtual learning scale [P < 0.04] indicating that applied sciences students encourage virtual classes and have a more positive opinion about virtual learning than the students in different majors. Finally, a significant distinction is discovered in students’ perceptions within the major on the clarity of instructions in a virtual learning environment scale [P = 4.60E-05] indicating that the field sciences experience more problems with addressing questions and gaining the required knowledge.

Table 5 presents the ANOVA test results based on age, the P-value scale exceeds 0.05 on the interaction between students, classmates, and their instructors. This indicated that there are no significant differences on this scale based on the students’ age for students’ perception of virtual teaching effectiveness compared to traditional e-techno which supports the study hypothesis for this scale.

Furthermore, the mean value results can be considered within the same category as the general statistical results in Table 2 where all ages are closer to agree on the interaction between students, classmates, and their instructor’s scale. Also, all ages are closer to no-preferences (except students aged between 29 and 33 and students aged more than 33, who are closer to agree) on the communication problems with classmates and the instructor. Moreover, all ages are closer to undecided (except students aged between 29 and 33 and students older than 33, who are closer to agree) on the difficulty of course material and learning expectations. Moreover, all ages are closer to no-preferences (except students aged between 29 and 33 who are closer to strongly agree and students aged more than 33 who are closer to agree) on the future courses in virtual learning. Finally, all ages are closer to undecided (except students aged between 29 and 33 who are closer to strongly agree and students aged more than 33 who are closer to agree) on the clarity of instructions in a virtual learning environment.

A significant difference was detected in students’ perceptions within the age of interaction between students and their classmates and their teacher’s scale [P < 0.02] indicating that students aged 29 years and above have more interaction issues in virtual e-techno than those below 29. Furthermore, a significant distinction is discovered in students’ perceptions within age on the communication problems with classmates and the instructor scale [P = 1.0E-06] indicating that students aged 29 and above face fewer problems related to communication during the virtual sessions than those below 29. Moreover, a significant distinction is discovered in students’ perceptions within age on the difficulty of course material and expectations of learning scale [P < 0.02] indicating that students aged 29 and above experience fewer problems related to learning and gaining knowledge during the virtual sessions than those below 29. In addition, a significant distinction is discovered in students’ perceptions of future courses on the virtual learning scale [P = 7.37E-04] indicating that students aged 29 and above encourage virtual e-techno and have a more positive opinion about virtual learning than those below 29. Finally, a significant distinction is discovered in students’ perceptions within age on the clarity of instructions in a virtual learning environment scale [P = 4.00E-06] indicating that students with age below 29 experience more problems with addressing questions and gaining the required knowledge, as compared with those above 28, during virtual sessions as compared with the traditional in-class learning.

Table 6 provides the ANOVA Test results based on country. The majority (97.3%) of participants are belonging to Middle Eastern countries; Palestine, Jordan, Saudi Arabia, and Egypt. The remaining 2.7% are from other countries including Bahrain, Lebanon, UK, Turkey, Algeria, Afghanistan, and Canada. Results show that the P-value exceeds 0.05 on the difficulty of course material and expectations of learning scale indicating that there are no significant differences on this scale which supports the study hypothesis for this scale. Furthermore, the mean value results can be considered within the same category as the general statistical results where students from all locations are closer to agreeing on the interaction between students, classmates, and their instructor’s scale. Also, all ages are closer to be unsure (except students located in Egypt, who are closer to disagreeing) on the communication problems with classmates and the instructor. Moreover, students from all locations are closer being imprecise on the difficulty of course material and learning expectations as well as on the future courses of virtual learning, which indicates the foggy situation within our coming schooling systems and implications.

**Table 6:** ANOVA test outcomes based on country

Dimension Scales	Country	N	Mean	Std.	F-value	P-value
D1	Egypt	254	3.86	0.84	5.37	2.8E-04
	Jordan	91	3.70	0.93		
	Palestine	286	4.08	0.82		
	Saudi Arabia	125	3.79	0.92		
	Others	21	3.62	1.21		
D2	Egypt	254	2.40	1.04	15.15	6.2E-12
	Jordan	91	2.85	1.19		
	Palestine	286	2.68	1.06		
	Saudi Arabia	125	3.28	1.22		
	Others	21	3.24	1.15		
D3	Egypt	254	2.97	0.82	0.63	0.64
	Jordan	91	2.92	0.99		
	Palestine	286	3.05	0.84		
	Saudi Arabia	125	2.99	0.88		
	Others	21	2.91	0.78		
D4	Egypt	254	2.87	1.139	4.24	2.1E-03
	Jordan	91	3.02	1.277		
	Palestine	286	3.27	1.114		
	Saudi Arabia	125	3.05	1.214		
	Others	21	3.29	0.956		
D5	Egypt	254	2.62	1.128	12.78	4.4E-10
	Jordan	91	2.88	1.263		
	Palestine	286	3.2	1.177		
	Saudi Arabia	125	3.34	1.224		
	Others	21	3.57	0.87		

Also, a significant distinction is discovered in students’ perceptions based on the country they are located in on the communication problems with classmates and the instructor scale [P = 6.26E-12] indicating that students located in Egypt face more problems related to communication during the virtual sessions than those in other countries. Moreover, a significant distinction is discovered in students’ perceptions based on the country they are located in on the future courses in virtual learning scale [P = 2.13E-03] indicating that students located in Egypt tend to discourage virtual e-techno and have a less positive opinion about virtual learning than those in other countries.

Table 7 displays the ANOVA test findings for students’ perceptions of virtual teaching effectiveness against traditional teaching, according to whether the department/faculty offered virtual courses prior to the COVID-19 outbreak.

**Table 7:** Based on supporting VC, ANOVA test outcomes

Dimension Scales	Supporting VC	N	Mean	Std.	F-value	P-value
D1	Yes	335	4.00	0.79	8.27	0.004
	No	442	3.82	0.93		
D2	Yes	335	2.93	1.10	19.55	1.1E-05
	No	442	2.57	1.14		
D3	Yes	335	3.16	0.80	22.89	2.0E-06
	No	442	2.87	0.88		
D4	Yes	335	3.28	1.13	18.49	0.0E + 00
	No	442	2.92	1.17		
D5	Yes	335	3.35	1.12	50.74	2.4E-12
	No	442	2.74	1.21		

As shown in Table 7, On all scales, the P-value is less than 0.05, showing that there are substantial variances within each scale in terms of supporting VCs for students’ perception of virtual teaching effectiveness which contradicts the study hypothesis.

A significant distinction is discovered in students’ perceptions in accordance of supporting VCs on the interaction between students, classmates, and their instructor’s scale [P = 0.004] indicating that students study in departments that support VCs are more concerned with the importance of interactions in virtual e-techno as compared to those who study in departments that do not support VCs. Furthermore, a significant distinction is discovered in students’ perceptions of supporting VCs on the communication problems with classmates and the instructor scale [P = 1.10E-05] indicating that students study in departments that do not support VCs facing more problems related to communications during the virtual sessions. Moreover, a significant distinction is discovered in students’ perceptions in accordance with supporting VCs on the difficulty of course material and expectations of learning scale [P = 2.00E-06] indicating that students study in departments that do not support VCs are experiencing fewer problems related to learning and absorbing knowledge during the virtual sessions as compared to those who study in departments that support VCs. In addition, a significant distinction is discovered in students’ perceptions in accordance of supporting VCs on future courses in a virtual learning scale [P = 0.00E + 00] indicating that students study in departments that do not support VCs tend to discourage virtual e-techno and have less positive opinion compared to those who study in departments that support VCs. Finally, a significant distinction is discovered in students’ perceptions in accordance with supporting VCs on the clarity of instructions in a virtual learning environment scale [P = 2.41E-12] indicating that students studying in departments that do not support VCs are experiencing more problems with addressing questions and gaining the required knowledge, than those who study in departments that support VCs, during virtual sessions as compared with the traditional sessions.

Table 8 displays the ANOVA test results for students' opinions of virtual teaching effectiveness against traditional teaching based on the academic year level of the students.

**Table 8:** ANOVA test results based on academic year

Dimension Scales	Academy year	N	Mean	Std.	F-value	P-value
D1	1st year	115	3.92	0.87	0.41	0.80
	2nd year	238	3.95	0.92		
	3rd year	169	3.86	0.84		
	4th year	183	3.87	0.83		
	5th year	72	3.88	0.96		
D2	1st year	115	3.07	1.13	8.84	5.5E-07
	2nd year	238	2.89	1.14		
	3rd year	169	2.70	1.13		
	4th year	183	2.42	1.05		
	5th year	72	2.42	1.13		
D3	1st year	115	2.92	0.85	1.49	0.20
	2nd year	238	3.08	0.82		
	3rd year	169	3.03	0.88		
	4th year	183	2.90	0.84		
	5th year	72	2.97	0.94		
D4	1st year	115	3.13	1.232	3.43	0.01
	2nd year	238	3.22	1.141		
	3rd year	169	3.13	1.132		
	4th year	183	2.92	1.133		
	5th year	72	2.74	1.207		
D5	1st year	115	3.23	1.285	10.28	4.1E-08
	2nd year	238	3.29	1.186		
	3rd year	169	2.93	1.14		
	4th year	183	2.74	1.112		
	5th year	72	2.5	1.245		

As shown in Table 8, the P-value exceeds 0.05 on two scales out of five; interaction between students, classmates, and their instructor’s scale, the difficulty of course material, and expectations of learning scale. It shows that there are no significant differences on these scales based on students’ academic year for students’ perception of virtual teaching effectiveness compared to traditional in-class teaching. The result supports the study hypothesis for these two scales. Furthermore, all mean value results can be considered within the same category as the general statistical results shown by Table 2 (except 4th and 5^th^-year students who are closer to disagreeing than no-preferences on the communication problems with classmates and the instructor scale). A significant distinction is discovered in students’ perceptions within the academic year on the communication problems with classmates and the instructor scale [P = 5.55E-07] indicating that students in the 4th and 5th academic years face more communication problems during the virtual sessions as compared to other students.

In addition, a significant distinction is discovered in students’ perceptions within the academic year on the future courses in virtual learning scale [P < 0.01] indicating that students in the 4th and 5th academic year have a less positive opinion about virtual learning than other students. Finally, a significant distinction is discovered in students’ perceptions within the academic year on the clarity of instructions in a virtual learning environment scale [P = 4.10E-08] indicating that students in the 4th and 5th academic years are experiencing more problems with addressing questions and gaining the required knowledge, than other students, during virtual sessions as compared with the traditional sessions.

Based on the student degree, Table 9 provides the ANOVA test results for students' opinions of virtual teaching efficacy compared to traditional teaching.

**Table 9:** The outcomes of the ANOVA test based on degree

Dimension Scales	Degree	N	Mean	Std.	F-value	P-value
D1	Diploma	70.00	4	1.00	0.29	0.83
	Bachelor	697.00	4	0.87		
	Master	9.00	4	0.90		
	Ph.D.	1.00	4	–		
D2	Diploma	70.00	3	1.07	5.82	6.2E-04
	Bachelor	697.00	3	1.14		
	Master	9.00	4	0.33		
	Ph.D.	1.00	4	–		
D3	Diploma	70.00	3	0.89	3.50	0.02
	Bachelor	697.00	3	0.85		
	Master	9.00	4	0.77		
	Ph.D.	1.00	4	–		
D4	Diploma	70.00	3.36	1.135	2.44	0.06
	Bachelor	697.00	3.04	1.166		
	Master	9.00	3.56	0.982		
	Ph.D.	1.00	4	–		
D5	Diploma	70.00	3.44	1.175	5.30	1.2E-03
	Bachelor	697.00	2.95	1.205		
	Master	9.00	3.89	0.601		
	Ph.D	1.00	3	–		

As shown in Table 9, the P-value exceeds 0.05 on two scales out of five; interaction between students, classmates, and their instructor’s scale, and difficulty of course material and expectations of learning scale. It shows no significant differences on these scales based on students’ degrees for students’ perception of virtual teaching effectiveness compared to traditional in-class teaching. The results support the study hypothesis for these two scales.

Furthermore, the mean value results can be considered within the same category as the general statistical results shown in Table 2 where students from all degrees are agreed on the interaction between students, classmates, and their instructor’s scale. Also, students from all degrees are closer to undecided (except master and Ph.D. students, who agree) on the communication problems with classmates and the instructor. Additionally, students from all degrees are closer to no-preferences (except master and Ph.D. students, who agree) on the difficulty of course material and learning expectations. Moreover, students from all degrees are closer to undecided (except master students who are closer to agree and Ph.D. students, who agree) on the future courses in virtual learning. Finally, students from all degrees are closer to no-preferences (except master, who are closer to agree) on the clarity of instructions in a virtual learning environment.

A significant difference is detected in students’ perceptions within students’ degree levels on the communication problems with classmates and the instructor scale [P = 6.20E-04] indicating that master and Ph.D. students are facing more problems related to communications during the virtual sessions as compared to other students. Moreover, a significant difference is detected in students’ perceptions within student’s degree levels on the difficulty of course material and expectations of learning scale [P < 2] indicating that master and Ph.D. students are experiencing fewer problems related to learning and absorbing knowledge during the virtual sessions as compared to other students. Finally, a significant difference is detected in students’ perceptions within student’s degree levels on the clarity of instructions in a virtual learning environment scale [P = 1.28E-03] indicating that master students are experiencing more problems with addressing questions and gaining the required knowledge, than other students, during virtual sessions as compared with the traditional sessions.

Table 10 displays the results of an ANOVA test for students' opinions of virtual teaching effectiveness against traditional teaching dependent on the type of device used during virtual sessions. As shown in the table, the P-value results exceed 0.05 on all scales except the communication problems with classmates and the instructor scale which indicates that there are no significant differences on these scales based on devices used by students during virtual learning sessions. The result supports the study hypothesis. Furthermore, a significant distinction is discovered in students’ perceptions based on the type of devices used during the virtual sessions on the communication problems with classmates and the instructor scale [P = 2.9E-03] indicating that students who used other devices than the stated in the above table are facing more problems related to communications during the virtual sessions as compared to other students.

**Table 10:** ANOVA test outcomes based on devices

Dimension Scales	Devices	N	Mean	Std.	F-value	P-value
D1	Desktop	51	3.9	0.78	0.65	0.58
	Laptop	359	3.87	0.85		
	Mobile phone	358	3.94	0.90		
	Others	9	3.67	1.39		
D2	Desktop	51	3.02	1.10	4.70	2.9E-03
	Laptop	359	2.834	1.15		
	Mobile phone	358	2.564	1.11		
	Others	9	2.722	1.39		
D3	Desktop	51	3.11	0.94	1.44	0.23
	Laptop	359	3.02	0.86		
	Mobile phone	358	2.97	0.83		
	Others	9	2.52	1.03		
D4	Desktop	51	3.23	1.254	1.40	0.24
	Laptop	359	3.09	1.202		
	Mobile phone	358	3.05	1.113		
	Others	9	2.39	1.024		
D5	Desktop	51	3.29	1.27	2.05	0.11
	Laptop	359	3.06	1.221		
	Mobile phone	358	2.91	1.182		
	Others	9	2.78	1.093		

Table 11 summarizes the P-value results across all factors for the five-dimension scales differently than the posted analysis of community question‐answering issues via machine learning and deep learning [28]. Our research considered the focus on general students’ perceptions of virtual teaching efficacy compared to traditional in-class teaching (highlighted in bold), as revealing significant differences in 67.5% of P-values, whereas 32.5% of P-values show no significant differences. These results can be a starting strategy for more in depth community investigation relating current problems to e-education, coming generation schooling and imminent technological improvement analysis to come.

**Table 11:** The P-value results for the five-dimension scales in all factors (summary table)

Dimension Scales	Factors				
	D1	D2	D3	D4	D5
Gender	**2.2E-07**	**6.5E-05**	0.83	0.36	0.17
Major	**0.02**	**0.02**	**3.0E-04**	**0.04**	**4.6E-05**
Age	0.18	**1.0E-06**	**0.01**	**7.3E-04**	**4.0E-06**
Country	**2.8E-04**	**6.2E-12**	0.64	**2.1E-03**	**4.4E-10**
Supporting VCs	**0.004**	**1.1E-05**	**2.0E-06**	**0.0E + 00**	**2.4E-12**
Academic year	0.80	**5.5E-07**	0.20	**0.01**	**4.1E-08**
Degree	0.83	**6.2E-04**	**0.02**	0.06	**1.2E-03**
Devices used	0.58	**2.9E-03**	0.23	0.24	0.11

Bold value indicates a statistically significant difference at α=0.05

## Conclusions

This study targets students’ perceptions of virtual learning during the COVID-19 pandemic. Five-dimension scales are used to analyze data collected from responses of 777 students from different countries, ages, degrees, and others. The dimension scales are the interaction between students, classmates and their instructors; communication problems with classmates and the instructor; the difficulty of course material and expectations of learning; future courses in virtual learning; and clarity of instructions in a virtual learning environment. Furthermore, seeking to prove that students’ perception of virtual teaching effectiveness compared to traditional in-class teaching is not affected by diversity, this research studies the effects based on eight factors, including gender, major, age, country, academic year level, academic degree, device types, and whether the department/college offered virtual courses prior to the outbreak of COVID-19.

The P-values in the majority of the results (67.5%) show a significant effect on students' perceptions of the effectiveness of virtual versus traditional in-class teaching, which contradicts the study's hypothesis. Conversely, 32.5% of P-values in all factors showed no significant differences in students' perceptions of the effectiveness of virtual teaching compared to standard in-class teaching. Furthermore, the P-values for some of the factors show that 100% of the dimension scales have significant differences including; major and whether the department/college offered virtual courses prior to the outbreak of COVID-19.

Based on our study, we concluded that: during virtual sessions as compared with traditional in-class learning female students face more problems with addressing questions and interacting with instructors than male students, and students who study in departments that do not support VCs can learn and gain knowledge more than those who study in departments that support VCs. However, more attention must be paid to improving virtual learning techniques and exploring alternative technologies to be ready for any unforeseen crisis in the future. Future studies will be focused on analyzing teachers’ perceptions regarding the virtual teaching process with respect to the findings of the current study to improve and refinement of future e-technical processes. Finally, we hope this research provides valuable points for researchers in various fields and interests in their future studies.

## Data Availability

Data sharing is not applicable to this article as no theoretical datasets were generated or analyzed during the current study.
